# In Situ Synthesis of Vertical Standing Nanosized NiO Encapsulated in Graphene as Electrodes for High‐Performance Supercapacitors

**DOI:** 10.1002/advs.201700687

**Published:** 2017-12-27

**Authors:** Jinghuang Lin, Henan Jia, Haoyan Liang, Shulin Chen, Yifei Cai, Junlei Qi, Chaoqun Qu, Jian Cao, Weidong Fei, Jicai Feng

**Affiliations:** ^1^ State Key Laboratory of Advanced Welding and Joining Harbin Institute of Technology Harbin 150001 China; ^2^ Key Laboratory of Functional Materials Physics and Chemistry of the Ministry of Education Jilin Normal University Siping 136000 China

**Keywords:** graphene, in situ synthesis, NiO, plasma‐enhanced chemical vapor deposition (PECVD), supercapacitors

## Abstract

NiO is a promising electrode material for supercapacitors. Herein, the novel vertically standing nanosized NiO encapsulated in graphene layers (G@NiO) are rationally designed and synthesized as nanosheet arrays. This unique vertical standing structure of G@NiO nanosheet arrays can enlarge the accessible surface area with electrolytes, and has the benefits of short ion diffusion path and good charge transport. Further, an interconnected graphene conductive network acts as binder to encapsulate the nanosized NiO particles as core–shell structure, which can promote the charge transport and maintain the structural stability. Consequently, the optimized G@NiO hybrid electrodes exhibit a remarkably enhanced specific capacity up to 1073 C g^−1^ and excellent cycling stability. This study provides a facial strategy to design and construct high‐performance metal oxides for energy storage.

## 1 Introduction

Supercapacitors, including electrical double‐layer capacitors (EDLCs) and pseudocapacitors, have become a research focus recently owing to their desirable properties, such as fast charge and discharge, high power densities, excellent cycling performance and safety.^1^, ^2^, ^3^, ^4^, ^5^ Compared with carbon nanomaterials for EDLCs, pseudocapacitor materials, especially transition metal oxides (TMOs), can provide higher specific capacity and energy density due to the efficient reversible redox reaction.^6^, ^7^, ^8^, ^9^ Among various TMOs, nickel oxide (NiO) has attracted great interest owing to the merits of low cost, high theoretical capacity and environmental friendliness.^10^, ^11^, ^12^, ^13^ Unfortunately, in many case, NiO often suffers from low specific capacity, inferior rate performance and poor cycling stability. It is mainly constrained by the poor conductivity, limited electroactive sites and structural instability during charge–discharge process.^11^, ^12^, ^13^

In order to solve these problems, various approaches have been provided, such as constructing nanoscale electrode materials, hybridizing with carbonaceous naomaterials, and doping with foreign atoms.^11^, ^14^, ^15^, ^16^ As one typical strategy, constructing nanoscale NiO electrode materials can enlarge the surface areas, such as nanoparticles, nanowires, and nanosheets.^16^, ^17^, ^18^ Nevertheless, the poor cycling performance, limited electroactive sites and inherently low conductivity of nanoscale NiO materials still need to be solved.^11^, ^12^ To address these problems, one effective strategy is to hybridize with carbonaceous materials, such as amorphous carbon, graphene, and carbon nanotubes, which can improve the conductivity and improve the structural stability.^19^, ^20^, ^21^ For example, Feng et al. synthesized peapod NiO nanoparticles in carbon fibers, which shows the high specific capacitance and good cycling stability.^15^ However, due to the aggregation and limited mutual connections, traditional approaches to hybridizing carbon nanomaterials with NiO often suffer from longer ion diffusion pathways and higher contact resistance.^21^, ^22^

In addition, another approach is to dope with foreign atoms.^11^, ^16^ Doping metallic Ni in NiO can show the much enhanced conductivity and faster charge transport during the redox reaction, leading to much improved electrochemical performances.^23^, ^24^ For example, Li et al. synthesized Ni@NiO core–shell nanoparticles tube arrays using ZnO template‐assisted electrodeposition approach, which showed enhanced specific capacitance up to about 950 F g^−1^ and better cycling stability.^16^ Lai et al. synthesized mesostructured NiO/Ni composites with 3D porous structure at mesoscale, which exhibited the highest capacitance up to 1305 F g^−1^ (522 C g^−1^) and maintained the good cycling stability.^11^ However, in most cases, these approaches often involve the addition of conducting polymer, which generally decreases the ion diffusion and electronic conductivity between electrodes and current collectors.^25^, ^26^, ^27^, ^28^ It is important to in‐situ synthesize 3D electrodes without organic binders, which can achieve the high specific capacitance and low contact resistance. For example, Mai and co‐workers designed and synthesized 3D Co_3_O_4_‐graphene@Ni*_x_*Co_2_ *_x_*(OH)_6_ *_x_* electrode directly on Ni foam, which delivered the high specific capacitance and good cycling stability.^29^

**Figure 1:**
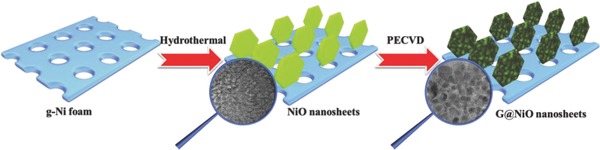
Schematic illustration of the synthesis process of G@NiO nanosheet arrays.

Based on above discussions, it is urgently needed to synthesize sophisticated nanomaterials, especially with designed and fabricated unique structure. Here, we design and synthesize novel vertical‐standing nanosized NiO encapsulated by high‐quality graphene layers (G@NiO) directly on conductive substrates as electrodes for high‐performance supercapacitors, as shown in **Figure** **1**. Briefly, NiO nanosheet arrays were firstly synthesized on g‐Ni foam. Then, the obtained NiO nanosheets were changed into nanosized NiO particles encapsulated in graphene layers by plasma enhanced chemical vapor deposition (PECVD) process. Accordingly, the G@NiO hybrid composites have the following advantages: (1) The vertical‐standing G@NiO nanosheet arrays feature large surface areas, short ion diffusion path and low contact resistance, thus leading to fast reaction kinetics. (2) Nanosized NiO could contribute to the high utilization of abundant electroactive sites for energy storage. (3) NiO nanoparticles self‐assembled on 3D conductive graphene frameworks can enable better electronic conductivity, mechanical properties for electrochemical performance of hybrid G@NiO samples. Benefiting from the integrated structures of NiO nanoparticles, high‐quality graphene layers, and vertical‐standing nanosheets, the resultant G@NiO hybrid electrodes can exhibit a high specific capacity of 1073 C g^−1^, good rate capability, and excellent cycling performance. And, an asymmetric supercapacitor (G@NiO//NGH) using G@NiO as positive electrode and nitrogen‐doped graphene hydrogels (NGHs) as negative electrode can show a high energy density of 52.6 Wh kg^−1^ at the power density of 800 W kg^−1^, and good cycling stability.

## 2 Results and Discussion

**Figure 2:**
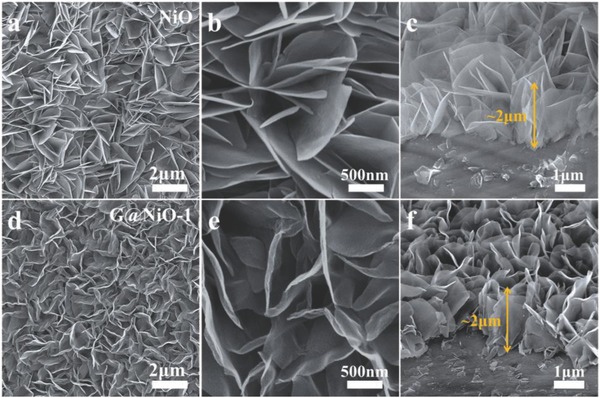
SEM images of a–c) NiO and d–f) G@NiO‐1 nanosheet arrays.

For comparison, the PECVD process was maintained for 0.5, 1, 2, and 3 min to obtain different samples (briefly named as G@NiO‐0.5, G@NiO‐1, G@NiO‐2, and G@NiO‐3). **Figure** **2** shows the typical scanning electron microscope (SEM) images of NiO and G@NiO‐1. As shown in Figure 2a,b, pristine NiO nanosheets could be synthesized on substrates. Notably, the nanosheets have a smooth surface with about 30 nm thickness and about 2 µm height (Figure 2b,c), where such high surface–volume ratio can provide sufficient surface areas with electrolytes. With the plasma treatment for 1 min, the resultant G@NiO‐1 nanosheets exhibit more flexible outer surface, as shown in Figure 2d,e. However, the vertical nanosheet morphology was still maintained and the height of G@NiO‐1 was also about 2 µm (Figure 2f). With extend the plasma exposing time, it can be found that the whole nanosheet morphology was maintained (Figure S2, Supporting Information).

**Figure 3:**
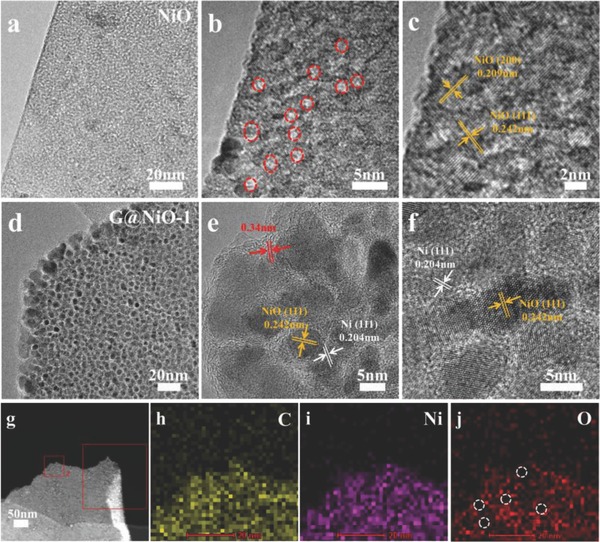
TEM and high‐magnification TEM images of a–c) NiO and d–f) G@NiO‐1 nanosheet arrays. g–j) STEM–EDS elemental analysis of G@NiO‐1 nanosheet arrays.

**Figure** **3**a,b exhibits the transmission electron microscope (TEM) images of NiO nanosheets. It can be found that the NiO nanosheet shows a mesoporous structure, which may be contributed to the evacuation of gaseous contents.^18^ Figure 3c shows that the lattice fringe shows an interplanar spacing of ≈0.209 and 0.242 nm, corresponding to the (200) and (111) planes of NiO phase.^12^, ^30^ After plasma treatment, the resultant G@NiO‐1 maintains the sheet‐like nanostructure, as shown in Figure 3d. Notably, it can be found that G@NiO‐1 nanosheets are changed into the unique core–shell nanoparticle structure, while these nanoparticles as core are connected by the outer shell layers, as shown in Figure 3d,e. Further, such core–shell structures are uniformly distributed across the whole nanosheet. As shown in Figure 3e,f, the interplanar spacings of ≈0.242 and 0.204 nm are corresponding to the (111) plane of NiO and (111) plane of Ni.^11^, ^12^ The formation of Ni mainly contributes to the reduction from H radical. Meanwhile, the shells in G@NiO samples are the highly crystalline, with about 0.34 nm interplanar spacing of graphene.^31^, ^32^ All obtained G@NiO samples with different plasma exposing time show such core–shell structures, in which NiO or Ni nanoparticles acts as the core and graphene layers acts as the shell (see Figure S3, Supporting Information). Further, scanning transmission electron microscopy (STEM) and energy dispersive spectrometer (EDS) elemental mapping images in Figure 3g–j show the uniform distributions of C, Ni, and O elements over the nanosheet.

**Figure 4:**
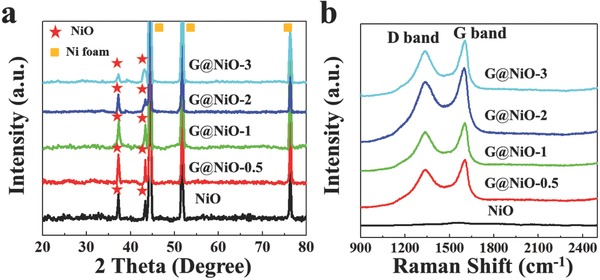
a) XRD and b) Raman characterizations of NiO and G@NiO nanosheet arrays.

The phase and composition of the G@NiO and NiO were characterized by X‐ray diffraction (XRD) and Raman, as shown in **Figure** **4**. As shown in Figure 4a, the peaks in all samples at 37.2° and 43.3° can be index to NiO (JCPDS no. 47‐1049), while other strong peaks can be index to Ni foam substrates Ni (JCPDS no. 04‐0850).^33^, ^34^ There are no obvious peaks of carbon materials in XRD patterns, mainly owing to the small amount of carbon materials produced by PECVD process. And Ni reduced from NiO is difficult to distinguish due to the existence of Ni foam substrates. Figure 4b shows two sharp peaks at about 1350 and 1580 cm^−1^, corresponding to the disorder induced D band and graphitic G band.^35^, ^36^ The sharp and high G band demonstrates the high crystallinity nature of graphene layers in all G@NiO samples.^37^, ^38^ Further, the intensity ratio of D band to G band (*I*_D_/*I*_G_) in all G@NiO samples is about 0.8, suggesting that low structural defects are introduced in graphene layers.^39^, ^40^ According to previous researches,^41^, ^42^ such thin graphene layers with high crystallinity nature are favorable for improving the electronic conductivity, mechanical properties, and electrochemical performance of hybrid core–shell G@NiO samples.

In addition, we also conduct X‐ray photoelectron spectroscopy (XPS) analysis to investigate the chemical states on the surface of G@NiO. In Figure S4 of the Supporting Information, the intensity of Ni is increased with the longer plasma exposition time, while the intensity for O^2−^ from NiO is gradually decreased. Further, we also calculate the ratio of NiO/Ni, according to the mass loading (see the Experimental Section in the Supporting Information). The calculated molar ratio of NiO/Ni is 93.6/6.4 for G@NiO‐0.5, 88.6/11.4 for G@NiO‐1, 75.3/24.7 for G@NiO‐2, and 65.7/34.3 for G@NiO‐3, respectively. A similar trend with XPS results can be also found. Undoubtedly, the longer plasma exposition time can produce more H radical from the decomposition of CH_4_. Thus, more Ni nanoparticles are produced and less NiO are remained. According to previous researches, metallic Ni shows no contributions on the capacitance.^11^, ^16^, ^43^ There is a trade‐off between metallic Ni and NiO with capacitive contribution. In the PECVD process, carbon gaseous sources (e.g., CH_4_, C_2_H_2_) provide the reactive radicals and play a predominant role in the nucleation and growth of graphene layers.^44^ Under this condition, H radical is inevitably produced and reacts with NiO, thus metallic Ni is produced. More importantly, the existence catalytic Ni is essential for the growth of high‐quality graphene layers.^45^, ^46^ Furthermore, high‐quality graphene layers with highly crystalline can enable better electronic conductivity, mechanical properties for electrochemical performance of hybrid core–shell G@NiO samples. Therefore, we believe such vertical standing G@NiO nanosheets can achieve better electrochemical properties.

**Figure 5:**
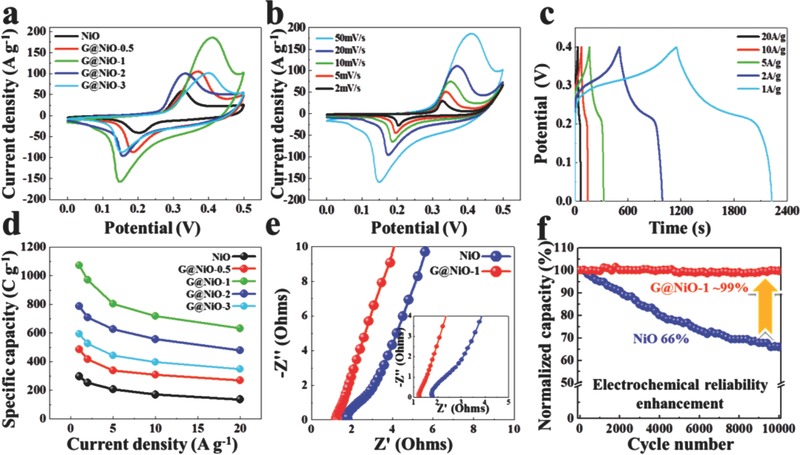
Electrochemical performances of G@NiO nanosheet arrays. a) CV comparison at the scan rate of 50 mV s^−1^. b) CV and c) GCD curves of G@NiO‐1. d) Specific capacity versus the current density for G@NiO nanosheet arrays. e) EIS spectra of the NiO and G@NiO‐1 electrodes. Inset is the local enlargement in the high‐frequency region. f) Cycling stability for 10 000 cycles at the current density of 10 A g^−1^ for NiO and G@NiO‐1.

The electrochemical performance of G@NiO and NiO are tested in a three‐electrode configuration using 2 m KOH. **Figure** **5**a shows the typical cyclic voltammetry (CV) curves of NiO and G@NiO electrodes at the scan rate of 50 mV s^−1^. Notably, G@NiO‐1 shows the largest enclosed CV curve area and the highest peak current among pristine NiO and other G@NiO samples, demonstrating the highest chare‐storage capacity.^47^ And CV curves of as‐synthesized NiO and G@NiO are shown in Figure 5b and Figure S5a–d (Supporting Information). Obviously, all samples show a pair of typical redox peaks, suggesting the typical battery‐type electrochemical behavior. It is mainly due to the faradaic redox reactions of Ni–O/Ni–O–OH.^48^, ^49^ Obviously, with increasing plasma exposure time from 0.5 to 3 min, more metallic Ni is produced and more graphene layers are encapsulated. Then, the conductivity of G@NiO samples is increased with the longer plasma exposure time. Thus, the overpotential of G@NiO samples is gradually reduced. The CV curves of G@NiO‐1 electrode at the scan rate of 2‐50 mV s^−1^ are shown in Figure 5b. There is a linear relationship between *v* ^1/2^ and cathodic peak current for NiO and G@NiO‐1, which indicates the good reversibility and the presence of diffusion‐controlled reactions in NiO and G@NiO‐1, as shown in Figure S6 of the Supporting Information. It suggests that the capacitance behavior in G@NiO‐1 is mainly come from the faradaic redox reactions.^50^, ^51^ In other words, G@NiO‐1 could be considered as the pseudocapacitive materials or battery‐like materials. From Figure S6 of the Supporting Information, we can find that the G@NiO‐1 composite represents faster diffusion velocity. According to the following equation

(1) $$i_{p}=\left(2.69\times 10^{5}\right)n^{3/2}AD_{0}^{1/2}C_{0}^{*}v^{1/2}$$

where *i*_p_, *n*, *A*, *D*_0_, *C*_0_ *, and *v* represent the peak current, number of electrons transfer, electrode area, diffusion coefficient, reactant concentration, and scan rate.^52^ With the assumption that NiO and G@NiO‐1 have the same *n*, *A*, and *C*_0_ * values, the diffusion coefficient of G@NiO‐1 is about eight times higher than that of NiO. It also demonstrates that such unique structure in G@NiO‐1 could increase the specific capacity and promote the charge transport during the faradaic redox reaction.

The galvanostatic charge–discharge (GCD) curves of all samples are shown in Figure 5c and Figure S5e–h (Supporting Information). The nonlinear GCD curves of the G@NiO‐1 electrode obtained at various current densities demonstrate the typical faradic behavior of the electrode.^32^, ^33^ The specific capacity of the as prepared pristine NiO and various G@NiO electrodes at different current densities is calculated from the GCD curves, as shown in Figure 5d. The specific capacity of the G@NiO‐1 electrode is calculated to be 1073 C g^−1^ at a current density of 1 A g^−1^, which is about three times higher than that of NiO (297 C g^−1^). Moreover, the capacity retention of the G@NiO‐1 electrode is about 59%, even at a high current density of 20 A g^−1^, demonstrating the good rate capability. As shown in Table S1 of the Supporting Information, it can be found that G@NiO‐1 electrode is competitive in current researches. The Nyquist plots of the NiO and G@NiO‐1 electrodes are shown in Figure 5e. Overall, both NiO and G@NiO‐1 electrodes exhibit the semicircle at the high frequency region and straight line in the low frequency region. It can be found that the spike‐like region in G@NiO‐1 is closer to the imaginary axis, suggesting the fast charge‐transfer kinetics and electric responses.^53^, ^54^, ^55^, ^56^ Further, the serial resistance (intercept on real axis) and charge transfer resistance (diameter of the semicircle) of the G@NiO‐1 is much lower than that of the NiO.^48^, ^50^, ^55^ The electrochemical impedance spectroscopy (EIS) data thus demonstrate that the G@NiO‐1 presents a much enhanced conductivity in both the serial and charge transfer parts, which is contributed by the tailored configuration of nanosized NiO and the high conductivity of graphene layers. Figure 5f shows the cycling performance of NiO and G@NiO‐1 electrodes at the current density of 10 A g^−1^. After 10 000 continuous cycles, the G@NiO‐1 electrode displays excellent cycling stability, with ≈99% retention of the initial capacity, which is much higher than about 66% capacitance retention of the pristine NiO electrode. In addition, Figure S7 of the Supporting Information shows the TEM images of NiO and G@NiO‐1 after 10 000 cycles. As shown in Figure S7a of the Supporting Information, it can be found that the structure of NiO has been destroyed with many holes. At the meantime, TEM results in Figure S7b,c of the Supporting Information indicate the G@NiO‐1 after 10 000 cycles remains the sheet‐like morphology consisting of the NiO nanoparticles encapsulated with the interconnected graphene layers. And Figure S7c of the Supporting Information also demonstrated that the metallic Ni retain the original state. In other word, the metallic Ni shows no contribution on the capacitance. The cycling tests further demonstrate that an interconnected graphene conductive network in G@NiO acts as binder to encapsulate the nanosized NiO particles as core–shell structure, which could prevent the structural collapse and electrode pulverization after cycling tests.

Such structure and morphology of G@NiO are similar to previous reported vertical graphene nanosheets (VFG).^57^, ^58^, ^59^, ^60^ Unlike the conventional hybrid approaches by integrating VFG with TMOs nanoparticles,^59^, ^60^ here we design and synthesize novel nanosized NiO particle encapsulated by graphene layer as the vertical standing nanosheet configuration. In order to further investigate the effect of graphene layers on the electrochemical performance, we also prepare the porous graphene (PG) by dissolving G@NiO‐1 sample in 3 m HCl for 24 h (see Figure S8, Supporting Information). As shown in Figure S8a of the Supporting Information, it can be found that graphene shells with highly crystalline still maintain the shell structures after corrosion. There are no remaining nanoparticles in the PG. In other words, the graphene shells are not fully encapsulated the NiO nanoparticles, which means that it would not hinder the infiltration of electrolyte ions. And the obtained PG only shows the limited specific capacity, suggesting that graphene layers in G@NiO mainly act conductive network on nanosized NiO particles. And PG could promote fast charge transport and maintain the structural stability. In addition, we also treated NiO samples under the plasma source of H_2_ and Ar for 1 min (NiO–Ni‐1, see Figures S9 and S10, Supporting Information). Electrochemical tests demonstrate NiO–Ni‐1 samples show the enhanced capacity, but the cycling stability of NiO–Ni‐1 is poor. Although metallic Ni could improve the conductivity, the poor interface connection between NiO and Ni in NiO–Ni nanosheet without organic binders would lead to the poor cycling stability. However, in our case, the existence catalytic Ni in G@NiO would help to the growth of high‐quality graphene layers by PECVD at the low temperature (350 °C). Further, such interconnected graphene conductive network with high quality can improve the electronic, mechanical, and electrochemical performances of G@NiO samples. More importantly, nanosized NiO particles with abundant eletroactive sites, which is beneficial for charge storage. In addition, vertical‐standing G@NiO nanosheets directly on the conductive substrates can provide a large contact area and reduce the contact resistance. Overall, by integrating these advantages of high‐quality interconnected graphene layer, nanosized NiO particles and vertical‐standing nanosheet structure, the G@NiO shows the high specific capacity, good rate capability, and excellent cycling stability.

**Figure 6:**
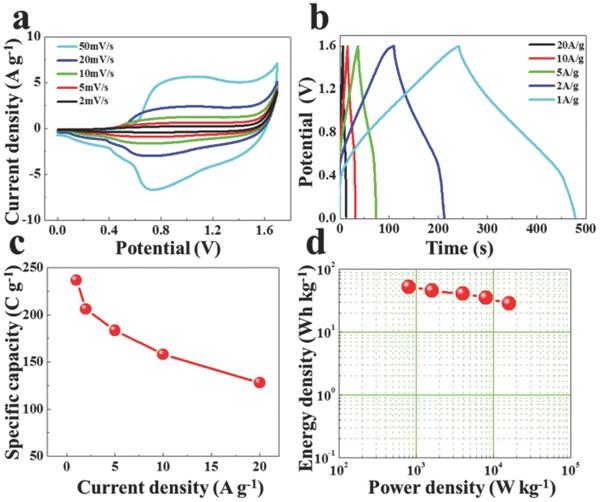
a) CV, b) CFG curves, c) the corresponding specific capacity, and d) Ragone plots of G@NiO‐1//NGH asymmetric device.

Currently, constructing the asymmetric supercapacitor is a promising strategy to improve the energy density of supercapacitors. For example, Mai and co‐workers synthesized a low‐crystalline iron oxide hydroxide nanoparticle anode with excellent electrochemical performance, and an asymmetric supercapacitor (ASC) device based on the low‐crystalline iron oxide hydroxide showed the high energy density.^61^ Here, an ASC device is fabricated by G@NiO‐1 as positive electrode and NGH (Figure S11, Supporting Information) as negative electrode. **Figure** **6**a shows the CV curves of the assembled G@NiO‐1//NGH device within an operating voltage window of 0–1.7 V. As shown in Figure 6a, there is a pair of apparent peaks at different scan rates, demonstrating the typical faradaic characteristics.^11^, ^47^, ^49^ As shown in Figure 6b, the GCD curves of G@NiO‐1//NGH device are almost symmetric at various current densities, suggesting the good Columbic efficiency.^47^, ^48^, ^49^ The specific capacity of the assembled G@NiO‐1//NGH device is calculated from the GCD curves, as shown in Figure 6c. The as‐fabricated G@NiO‐1//NGH device shows the specific capacity up to 237 C g^−1^ at 1 A g^−1^, and still retains 128 C g^−1^ at 20 A g^−1^, suggesting the good rate performance. Further, the G@NiO‐1//NGH device shows about 91.7% capacity retention after 5000 cycles (Figure S12, Supporting Information). Figure 6d shows the Ragone plot of as‐fabricated G@NiO‐1//NGH device. Notably, the as‐fabricated G@NiO‐1//NGH device shows a high energy density of 52.6 W h kg^−1^ at a power density of 800 W kg^−1^, which is much higher than that for ASC devices based on nickel‐based materials (Table S2, Supporting Information).

## 3 Conclusion

In conclusion, we provide a new approach to boost the electrochemical performance of NiO by constructing the unique structure of G@NiO composites without organic binders, where nanosized NiO particles are in situ encapsulated by high‐quality graphene layers as the vertical‐standing nanosheet structure. The optimized G@NiO hybrid electrodes shows an improved capacity of as high as 1073 C g^−1^, almost three times higher than that of the counterpart in pristine NiO, good rate capability, and excellent cycling performance. Further, the constructed G@NiO‐1//NGH device shows a maximum energy density up to 52.6 W h kg^−1^ at a power density of 800 W kg^−1^, and good cycling performance. In principle, the strategy in our study feasibly offers insight into the in situ forming graphene on nanosized TMOs particles directly as electrodes for maximizing their electrochemical performances, which may help to accelerate development of TMOs as electrode for high‐performance supercapacitors.

## Conflict of Interest

The authors declare no conflict of interest.

## Supporting information

Supplementary
