# Supplementary material for: In Situ Synthesis of Vertical Standing Nanosized NiO Encapsulated in Graphene as Electrodes for High‐Performance Supercapacitors
Source: Adv Sci (Weinh). 2017 Dec 27;5(3):1700687. doi: 10.1002/advs.201700687 (PMC5867121; doi:10.1002/advs.201700687)
Supplement: Supplementary file 1 — Supplementary [file ADVS-5-1700687-s001.pdf]

Copyright WILEY-VCH Verlag GmbH & Co. KGaA, 69469 Weinheim, Germany, 2017.

ADVANCED SCIENCE
Open Access

Supporting Information

for *Adv. Sci.,* DOI: 10.1002/advs.201700687

In Situ Synthesis of Vertical Standing Nanosized NiO Encapsulated in Graphene as Electrodes for High-Performance Supercapacitors

Jinghuang Lin, Henan Jia, Haoyan Liang, Shulin Chen, Yifei Cai, Junlei Qi, Chaoqun Qu, Jian Cao,* Weidong Fei, and Jicai Feng*

Supporting Information

In-situ synthesis of vertical standing nanosized NiO encapsulated in graphene as electrodes for high-performance supercapacitors

Jinghuang Lin, Henan Jia, Haoyan Liang, Shulin Chen, Yifei Cai, Junlei Qi, Chaoqun Qu, Jian Cao*, Weidong Fei and Jicai Feng*

Experimental

Synthesis of NiO nanosheets. Ni foam was cleaned by 1M HCl, deionized water and ethanol in the ultrasonic cleaner for several times. We conducted the chemical vapor deposition (CVD) process to synthesize thin graphene layers on the surface of Ni foam (g-Ni), which is according to Cheng *et al.* research.[1] After that, g-Ni foam was used as the conductive substrates for synthesizing NiO nanosheet arrays. Briefly, $Ni(NO_3)_2 \cdot 6H_2O$ (1 mmol) and urea (5 mmol) were added to 40 ml of deionized water to obtain a mixed solution. Then, g-Ni foam were transferred to the 50 ml reaction vessel and maintained at 120 ^oC for 6 h. The fabricated precursors were washed deionized water and ethanol for several times. The synthesized precursors were annealed at Ar atmosphere in 350 ^oC for 1 h to obtain NiO.[2,3] The mass loading of NiO was calculated by the weight difference before and after hydrothermal and annealing processes. The mass loading of NiO is about 1.60 mg cm^{-2}.

Synthesis of vertical standing nanosized NiO encapsulated in graphene (G@NiO). Plasma enhanced chemical vapor deposition (PECVD) enables growth of various carbon nanometerials on kinds of substrate without catalysts under the low temperature. In this case, we conducted PECVD process to encapsulate NiO by graphene. Firstly, the obtained NiO was heated to 350 ^oC at the Ar atmosphere. In the

next step, a mixture of CH_4 and Ar with a gas flow rate ratio of 5sccm/95sccm was introduced to the chamber. During this process, the chamber pressure was kept at 500 Pa and the plasma source was turned on at a power of 200W. And the plasma condition was maintained for 0.5min, 1min, 2min and 3min before cooling down the room temperature (briefly named as G@NiO-0.5, G@NiO-1, G@NiO-2 and G@NiO-3). For comparison, the pristine NiO samples were also treated under the plasma source of H_2 and Ar (10sccm/95sccm) for 1min (named as NiO-Ni-1). The mass loading of G@NiO samples was calculated by subtracting the weight before hydrothermal process from the weight after PECVD process. The mass loading of G@NiO-0.5, G@NiO-1, G@NiO-2 and G@NiO-3 is 1.59, 1.61, 1.64 and 1.65 mg cm^{-2}, respectively.

Materials characterization. The morphology and microstructure of obtained samples were investigated by scanning electron microscope (SEM, Helios Nanolab 600i) and transmission electron microscope (TEM, Tecnai G2 F30) equipped with energy dispersive X-ray analyzer (EDAX, AMTEK). X-ray diffraction (XRD, D/MAX 2200 VPC) was performed in the 2θ range from 20 to 80°. Furthermore, Raman spectrum (Renishaw-InVia) and X-ray photoelectron spectroscopy (XPS) (ESCALAB 250Xi) were also carried out.

Electrochemical tests. The electrochemical tests were conducted in the three-electrode configuration by CHI 760E and PARSTAT 4000A, using 2 M KOH as electrolyte. While the obtained sample was directly used as working electrode, Pt foil and Hg/HgO electrode were used as the counter and reference electrodes, respectively. Electrochemical impedance spectroscopy (EIS) was measured in the frequency range of 0.1 to 10^5 Hz with an amplitude of 5 mV. The specific capacitance (C_s) and specific capacity (C_m) of obtained samples was calculated by the following equations:

$$C_s = I\,\Delta t/\, m\Delta V \quad (1)$$

$$C_m = I\,\Delta t/\, m \quad (2)$$

where C, I, Δt, m, and ΔV mean the specific capacitance, the current, the discharging time, the mass loading of the active materials and the potential window, respectively.[3] In order to widen the potential window, the symmetric supercapacitor (ASC) device was assembled using G@NiO-1 as positive electrode and nitrogen-doped graphene hydrogels (NGH) as negative electrode. The synthesis process of NGH was according to previous research [4]. The energy density (E, Wh kg^{-1}) and power density (P, W kg^{-1}) of assembled ASC devices were calculated by the following equations[5]:

$$E = 1/2\, CV^2 \quad (2)$$

$$P = E\,\Delta t \quad (2)$$

where C, V and Δt mean the specific capacitance, potential window and discharging time in the ASC device. The energy density is calculated based the mass of active materials (total active material loadings of the G@NiO and NGH).

Mass loading calculations. The mass loading of graphene on the g-Ni foam was calculated by the weight difference before and after CVD process, using a high-precision balance (Denver Instruments, sensitivity: 0.01 mg). And ten samples with a size of 2 × 5 cm^2 were weighed. The graphene loading on g-Ni foam was calculated to be about 0.08mg cm^{-2}. In order to calculate the graphene layers on NiO nanosheets by PECVD process, the obtained G@NiO products were dissolved in 3M HCl for 24 h. After that, the obtain products were washed by water and ethanol for several times, and dried at 80 ^oC overnight. By weighing these products, the graphene layers on NiO can be estimated to about 0.03 mg cm^{-2} for G@NiO-0.5, 0.08 mg cm^{-2} for G@NiO-1, 0.13 mg cm^{-2} for G@NiO-2 and 0.17 mg cm^{-2} for G@NiO-3, respectively. And the mass loading of graphene layers on NiO was all subtracted the

mass loading of graphene on g-Ni substrate by CVD.

As for the ratio of NiO/Ni, we calculated the mass loading of NiO by annealing the G@NiO samples at 400 ^{o}C with the H_2/Ar (1:9) atmosphere for 2h. After the annealing process, the weight difference (m_1) can be calculated. Thus, the NiO content in G@NiO samples can be calculated on the reaction below

$NiO + H_2 = Ni + H_2O$ Δ

74.71 16.02

$74.71m_1/16.02$ (g) m_1 (g)

Assuming that there are m_0 (g) active materials in G@NiO sample, it should be firstly subtracted the mass loading (m_2) of graphene layers produced by PECVD process. Then, the mass loading of Ni can be estimated by the equation: $m_2=m_0-m_2-m_1/16.02$. Finally, the molar ratio of NiO/Ni can be estimated as following: $m_1/16.02 : m_2/58.69$. The calculated molar ratio of NiO/Ni is 93.6/6.4 for G@NiO-0.5, 88.6/11.4 for G@NiO-1, 75.3/24.7 for G@NiO-2 and 65.7/34.3 for G@NiO-3, respectively.

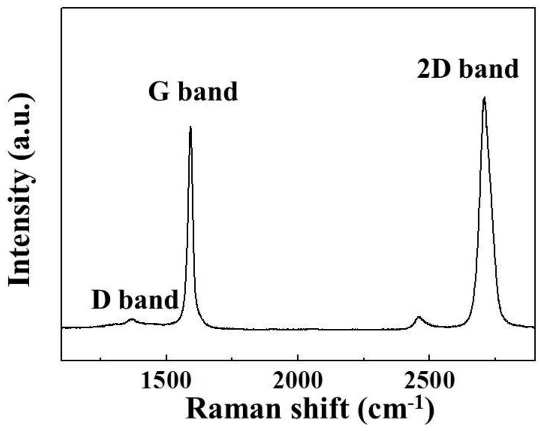

Figure S1. Raman spectrum of g-Ni foam

As shown in **Figure S1**, the Raman spectrum of g-Ni foam shows three major bands at about 1350 (D band), 1580 (G band) and 2700 cm^{-1} (2D band).[6] The D band is induced by the disordered carbon atoms, while G band represents the sp^2-hybridized graphitic carbon atoms.[7] The weak D band and high G band in g-Ni foam suggests that the graphene on Ni-foam shows the high degree of graphitization.[6,7]

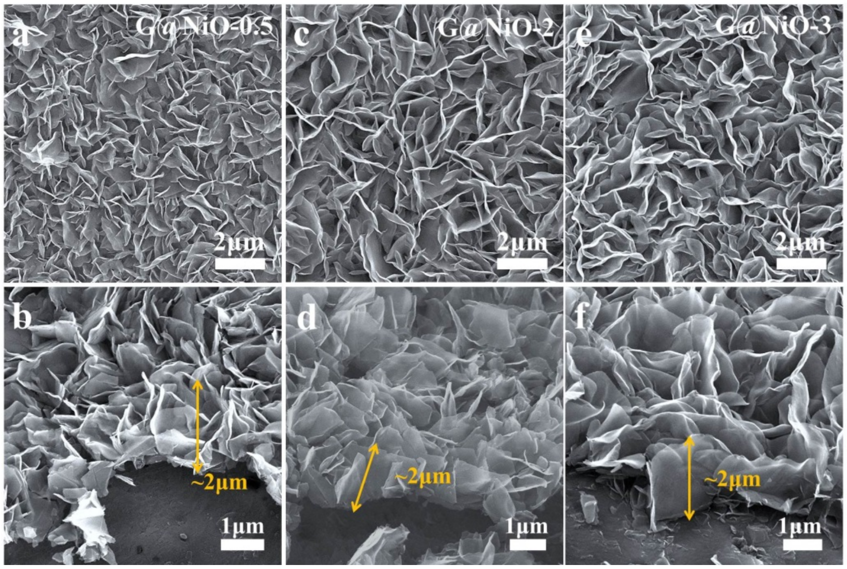

Figure S2. SEM images of (a,b) G@NiO-0.5, (c,d) G@NiO-2 and (e,f) G@NiO-3

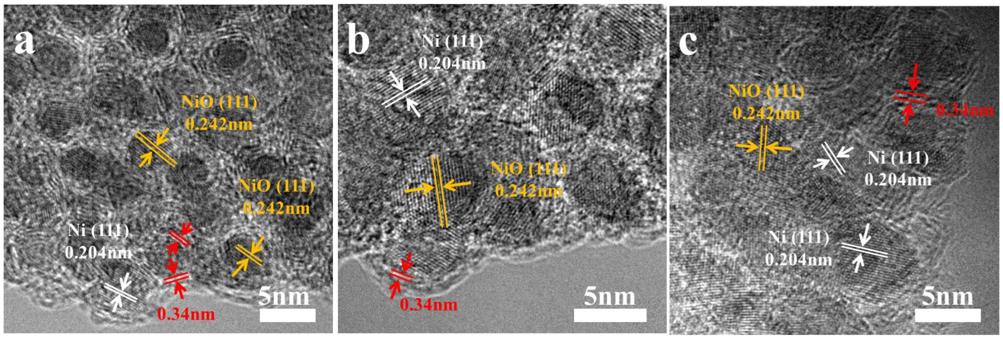

Figure S3. The HRTEM images of (a) G@NiO-0.5, (b) G@NiO-2 and (c) G@NiO-3

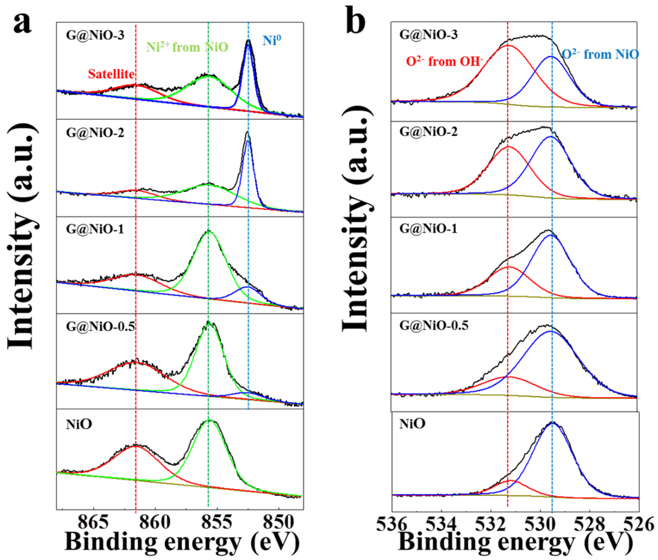

Figure S4. XPS spectra of (a) Ni $2p^{3/2}$ and (b) O 1s in G@NiO nanosheet arrays

As shown in **Figure S4a**, Ni $2p^{3/2}$ can be deconvoluted into three peaks at 861.6 eV, 855.7 eV and 852.7 eV, which corresponds to the satellite, Ni^{2+} from NiO and metallic Ni.[8,9] As shown in **Figure S4b**, O 1s can be deconvoluted into two peaks at the binding energies of 529.5 eV and 531.2 eV, which can be indexed to O^{2-} from NiO and O^{2-} form OH^{-}.[10,11] It can be found that the intensity of Ni was increased with the longer plasma exposition time, while the intensity for O^{2-} from NiO was gradually decreased.

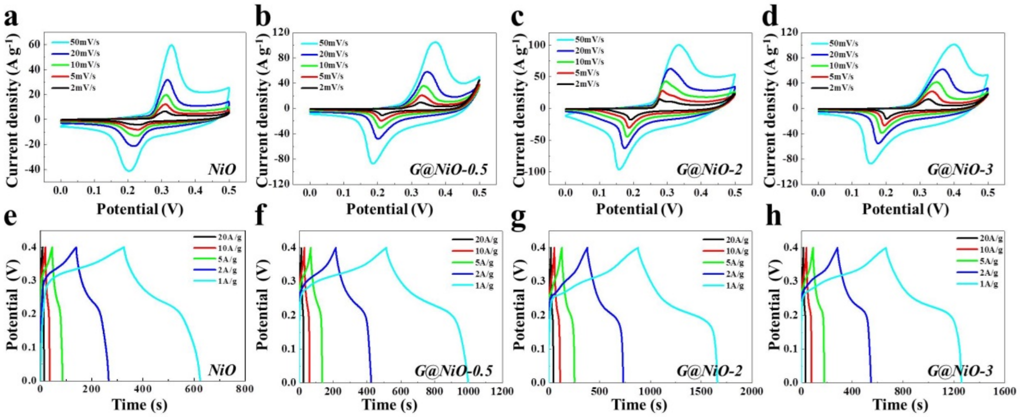

Figure S5. CV and GCD curves for (a, e) NiO, (b, f)G@NiO-0.5, (c, g) G@NiO-2 and (d, h) G@NiO-3 electrodes

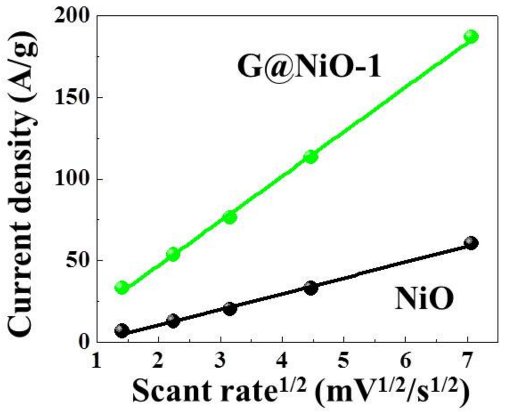

Figure S6. The corresponding current density (i)-$v^{1/2}$ (scant rate$^{1/2}$) plots in NiO and G@NiO-1 electrodes

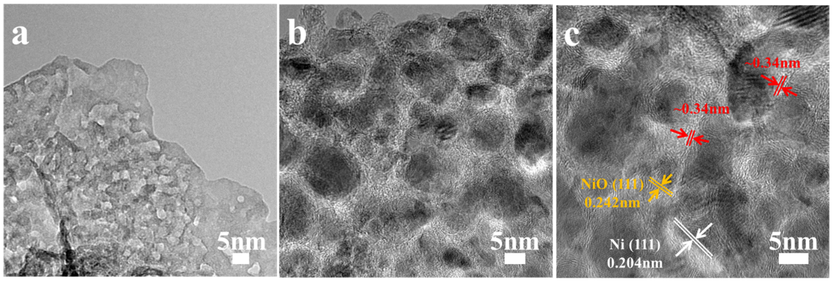

Figure S7. The HRTEM images for (a) NiO and (b, c) G@NiO after 10000 cycles

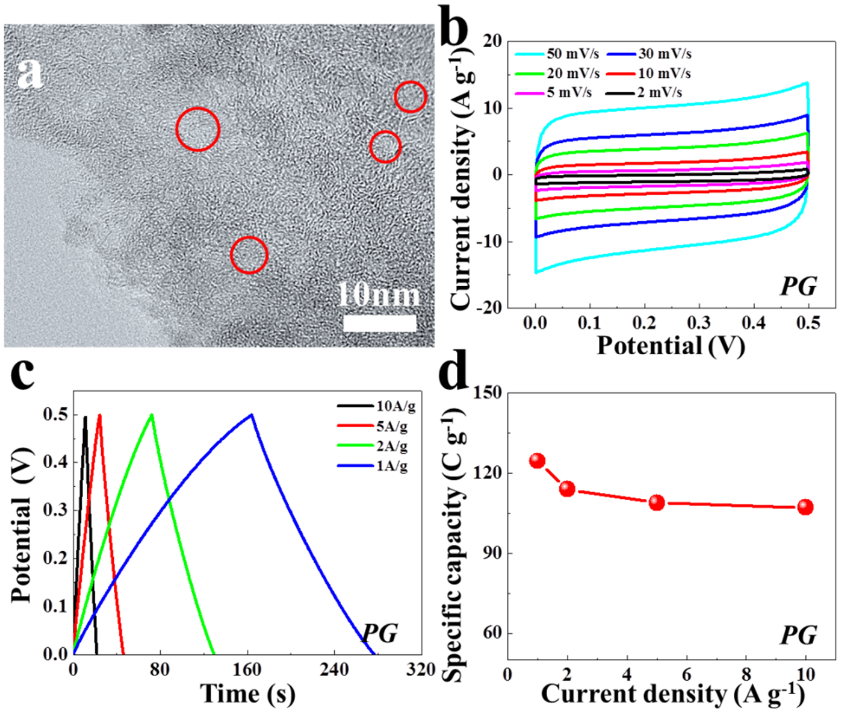

Figure S8. (a) The HRTEM image for PG. (b) CV and (c) GCD curves of PG. (d) Specific capacity versus the current density for PG.

In order to further investigate the effect of graphene layers on the electrochemical performance, we also prepare the porous graphene (PG) by dissolving G@NiO-1 sample in 3M HCl for 24 h (see **Figure S8**). As shown in **Figure S8a**, the PG shows the porous structures (markd with red cycles) after dissolving process. It suggests that the interconnected graphene do not fully encapsulate the nanoparticles. In addition, we also prepared the electrodes by mixing the PG (80% wt.%), acetylene black (10 wt.%) with polyvinylidene fluoride (10 wt.%) binder in N-methyl pyrrolidinone solvent. The mixed slurry were coated onto g-Ni foam and dried at 80 ^{o}C overnight. The electrochemical tests demonstrates that PG shows the limited specific capacity (only about 125 C g^{-1} at 1 A g^{-1}), which is much lower than that of G@NiO.

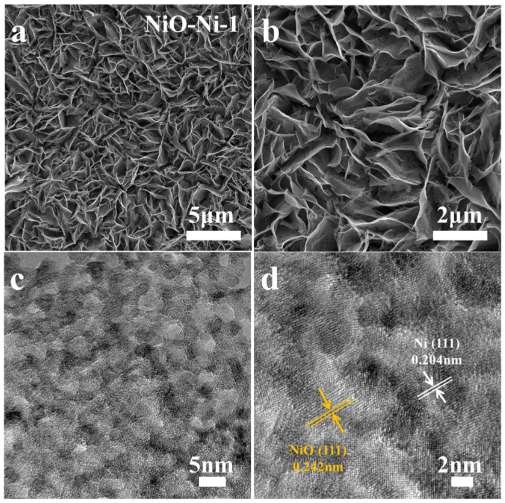

Figure S9. (a, b) SEM and (c, d) HRTEM images of NiO-Ni-1

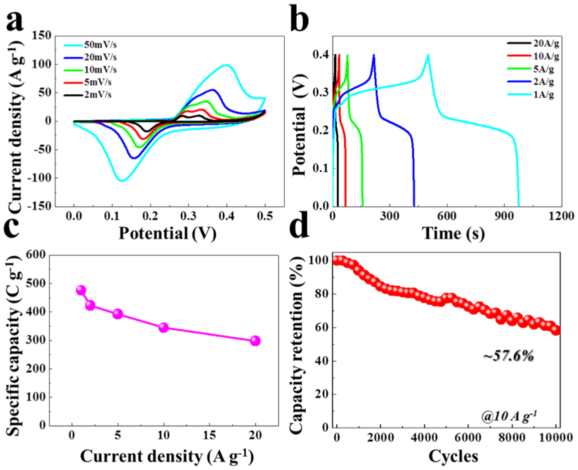

Figure S10. (a) CV and (b) GCD curves of NiO-Ni-1. (c) Specific capacity versus the current density for NiO-Ni-1. (d) Cycling stability for 10000 cycles at the current density of 10 A g^{-1} for NiO-Ni-1

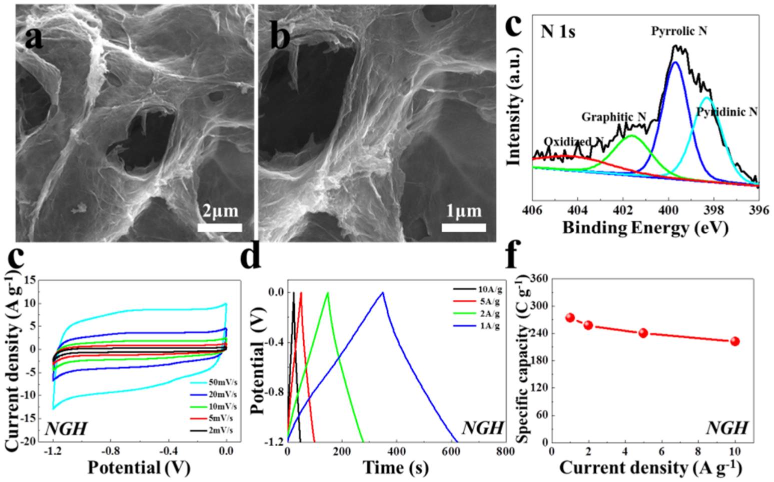

Figure S11. (a, b) SEM images of nitrogen-doped graphene hydrogels (NGH). (c) High-resolution N1s spectrum of NGH. (d) CV curves of NGH at various scan rate, (e) GCD curves of NGH at various current densities and (f) corresponding specific capacity

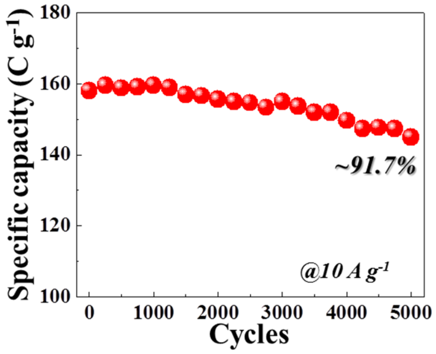

Figure S12. The cycling stability of G@NiO-1//NGH asymmetric device

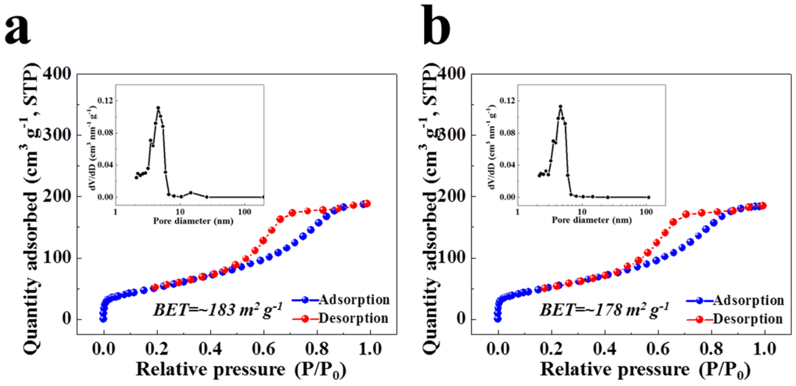

Figure S13. The N_2 adsorption/desorption isotherm of (a) G@NiO-1 and (b) NiO, and the inset is the corresponding pore size distribution

The specific surface area and pore size distributions of the G@NiO-1 and NiO were tested by the Brunauer-Emmet-Teller (BET) and Barrett-Joyner-Halenda (BJH) methods using a Micromeritics ASAP 2020. As shown in **Figure S13**, G@NiO-1 sample has the BET surface area of 183 m^2 g^{-1}, while pristine NiO sample has the value of 178 m^2 g^{-1}. The pores in both samples are mainly in the mesoporous range (about 5 nm).

Table S1. The specific capacitance of various electrodes in the three-electrode system in references

electrode materials	electrolyte	potential window	Specific capacity	reference
Ni@NiO core-shell nanoparticle tube arrays	1 M NaOH	0-0.5 V	500 C g^{-1}	9
hollow NiO elliptical microstructure	6 M KOH	0-0.45 V	715 C g^{-1}	12
Porous NiO nanosheets	6 M KOH	0-0.35 V	348 C g^{-1}	13
NiO/ITO nanowire heterostructures	1 M KOH	0-0.5 V	513 C g^{-1}	14
Hierarchical flower-like C/NiO composite hollow microspheres	2 M KOH	0-0.45 V	263 C g^{-1}	15
NiO/rGO	1 M KOH	0-0.5 V	295 C g^{-1}	16
NiO nanoparticles in mesoporous carbon nanosphere	6 M KOH	-1-0V	406 C g^{-1}	17
nickel-cobalt hydroxide/graphene	6 M KOH	0-0.4 V	667 C g^{-1}	18
NiO-In_2O_3 microflower	3 M KOH	-0.2-0.55V	767 C g^{-1}	19
ultrathin porous NiO nanoflake	1 M KOH	0-0.4 V	805 C g^{-1}	20
carbon-coated NiO/graphene	1 M LiOH	0-0.45 V	184 C g^{-1}	21
H-TiO_2@$Ni(OH)_2$/C fiber	6 M KOH	0-0.4 V	737 C g^{-1}	22
meso-NiO/Ni	2 M KOH	0-0.4 V	522 C g^{-1}	23
double-shelled tremella-like NiO@Co_3O_4@MnO_2	1 M KOH	-0.1-0.45 V	436 C g^{-1}	24
NiO/NiMn-layered double hydroxide nanosheet	3 M KOH	0-0.5 V	469 C g^{-1}	25
nanoporous Ni@NiO nanoparticles	6 M KOH	0-0.55 V	353 C g^{-1}	26
vertically aligned MoS_2-NiO hybrids	6 M KOH	0-0.5 V	540 C g^{-1}	27
carbon quantum dots/NiO	2 M KOH	0-0.5 V	665 C g^{-1}	28
NiO/perforated graphene nanosheet	1 M KOH	0-0.5 V	729 C g^{-1}	29
$NiGa_2O_4$ nanosheet	6 M KOH	0-0.5 V	754 C g^{-1}	30
3D Ni-Co-Mn oxide	6 M KOH	0-0.45 V	518 C g^{-1}	31
sandwich-like CNTs/$NiCo_2O_4$	2 M KOH	0-0.5 V	876 C g^{-1}	32
NiO mesoporous nanowall/rGO	3 M KOH	0-0.5 V	683 C g^{-1}	33
$NiCo_2O_4$/NiO nanosheets on SiC nanowires	6 M KOH	0-0.4 V	720 C g^{-1}	34
NiO nanostructured	2 M KOH	0-0.45 V	155 C g^{-1}	35
$Ni(HCO_3)_2$ nanosheets	3 M KOH	0-0.5 V	899 C g^{-1}	36
Ni-Co oxide@3D carbon skeleton	6 M KOH	0-0.5 V	813 C g^{-1}	37
$NiCo_2O_4$/CNT	6 M KOH	-0.05-0.45 V	340 C g^{-1}	38
$NiCo_2S_4$/MWCNTs	6 M KOH	0-0.4 V	832 C g^{-1}	39
Ni_3S_2 arrays	3 M KOH	0-0.4 V	420 C g^{-1}	40
$NiCo_2S_4$@$Ni_3V_2O_8$	2 M KOH	0-0.4 V	512 C g^{-1}	41
Ni-MOF superstructure	3 M KOH	0-0.4 V	395 C g^{-1}	42

multishelled NiO hollow microspheres	2 M KOH	0-0.45 V	576 C g^{-1}	43
NiO mesocrystals	2 M KOH	0-0.5 V	520 C g^{-1}	44
NiO encapsulated N-rich carbon hollow spheres	1 M KOH	0-0.5 V	565 C g^{-1}	45
G@NiO	2 M KOH	0-0.4 V	1073 C g^{-1}	This work

Table S2. Comparison of the electrochemical performance of as-fabricated ASC device with those in previous reports.

**Asymmetric supercapacitor**	**Energy density (Wh kg^{-1})**	**Corresponding Power density (W kg^{-1})**	**Reference**
nickel-cobalt hydroxide/graphene//AC	41	216	18
NiO//rGO	45.3	1081.9	20
meso-NiO/Ni//carbon nanocages	19.1	700	23
MoS_2–NiO//MoS_2–Fe_2O_3	39.6	807.2	27
$NiGa_2O_4$//Fe_2O_3	45.2	1600	30
$NiCo_2S_4$/MWCNTs//rGO	51.8	865	39
Ni_3S_2 arrays//N-porous graphitic carbon	48.5	87.4	40
$NiCo_2S_4$@$Ni_3V_2O_8$//AC	42.7	200	41
multishelled NiO hollow microspheres//RGO@Fe_3O_4	51.0	800	43
NiO mesocrystals//N-doped graphene	34.4	1500	44
NiO encapsulated N-rich carbon hollow spheres//N-doped graphene	50	740	45
MnCo-LDH@$Ni(OH)_2$//AC	47.9	750.7	46
$CuCo_2O_4$/NiO//AC	51.8	866	47
NiO//AC	43.5	2100	48
$MnCo_2O_4$@$Ni(OH)_2$//AC	48	1400	49
$ZnCo_2O_4$@$Ni_xCo_{2x}(OH)_{6x}$//AC	26.2	511.8	50
Ni-Co-S/graphene//carbon nanosheets	43.3	800	51
CC@Co_3O_4//CC@NC	41.5	6200	52
Co(P,S) nanotubes//CC	39	800	53
onion-like $NiCo_2S_4$ particles//AC	42.7	1583	54
G@NiO-1//NGH	52.6	800	This work

References

1. Z. Chen, W. Ren, L. Gao, B. Liu, S. Pei and H. Cheng, *Nat. Mater.* 2011, 10, 424.

2. S. Liu, S. C. Lee, U. M. Patil, C. Ray, K. V. Sankar, K. Zhang, A. Kundu, S. Kang, J. H. Park and S. C. Jun, *J. Mater. Chem. A* 2017, 5, 4543.

3. G. Meng, Q. Yang, X. Wu, P. Wan, Y. Li, X. Lei, X. Sun and J. Liu, *Nano Energy* 2016, 30, 831.

4. Y. Wang, Z. Wei, Y. Nie and Y. Zhang, *J. Mater. Chem. A* 2017, 5, 1442.

5. C. Young, R. R. Salunkhe, S. M. Alshehri, T. Ahamad, Z. Huang, J. Henzie and Y. Yamauchi, *J. Mater. Chem. A* 2017, 5, 11834.

6. H. Huang, L. Ma, C. S. Tiwary, Q. Jiang, K. Yin, W. Zhou and P. M. Ajayan, *Small 2017*, 13, 1603013.

7. K. N. Kudin, B. Ozbas, H. C. Schniepp, R. K. Prud'homme, I. A. Aksay and R. Car, *Nano Lett.* 2008, 8, 36.

8. S. Ci, Z. Wen, Y. Qian, S. Mao, S. Cui and J. Chen, *Sci. Rep.* 2015, 5, 11919.

9. Q. Li, C. Liang, X. Lu, Y. Tong and G. Li, *J. Mater. Chem. A* 2015, 3, 6432.

10. S. Liu, K. Hui, K. Hui, V. V. Jadhav, Q. X. Xia, J. M. Yun, Y. Cho, R. S. Mane and K. H. Kim, *Electrochim. Acta* 2016, 188, 898.

11. D. Li, Y. Gong, Y. Zhang, C. Luo, W. Li, Q. Fu and C. Pan, *Sci. Rep.* 2015, 5, 12903.

12. S. Wang, W. Li, L. Xin, M. Wu, W. Sun and X. Lou, *Chem. Eng. J.* 2017, 321, 546.

13. X. Sun, G. Wang, J. Hwang and J. Lian, *J. Mater. Chem.* 2011, 21, 16581.

14. Q. Lu, M. W. Lattanzi, Y. P. Chen, X. M. Kou, W. F. Li, X. Fan, K. M. Unruh, J. G. G. Chen and J. Q. Xiao, *Angew. Chem.* 2011, 50, 6847.

15. T. Liu, C. Jiang, B. Cheng, W. You and J. Yu, *J. Power Sources* 2017, 359, 371.

16. H. Kahimbi, S. B. Hong, M. Yang and B. G. Choi, *J. Electroanal. Chem.* 2017, 756, 14.

17. M. Liu, X. Wang, D. Zhu, L. Li, H. Duan, Z. Xu, Z. Wang and L. Gan, *Chem. Eng. J.* 2017, 308, 240.

18. Y. Chen, W. K. Pang, H. Bai, T. Zhou, Y. Liu, S. Li and Z. Guo, *Nano Lett.* 2017, 17, 429.

19. N. Padmanathan, H. Shao, D. MaNulty, C. O'Dwyer and K. M. Razeeb, *J. Mater. Chem. A* 2016, 4, 4820-4830.

20. S. Wu, K. S. Hui, K. N. Hui and K. H. Kim, *J. Mater. Chem. A* 2016, 4, 9113.

21. L. Yu, G. Wan, X. Peng, Z. Dou, X. Li, K. Wang, S. Lin and G. Wang, *RSC Adv.* 2016, 6, 14199.

22. Q. Ke, C. Guan, X. Zhang, M. Zheng, Y. Zhang, Y. Cai, H. Zhang and J. Wang, *Adv. Mater.* 2017, 29, 1604164.

23. H. Lai, Q. Wu, J. Zhao, L. Shang, H. Li, R. Che, Z. Lyu, J. Xiong, L. Yang, X. Wang and Z. Hu, *Energy Environ. Sci.* 2016, 9, 2053.

24. H. Wang, Q. Ren, D. J. L. Brett, G. He, R. Wang, J. Key and S. Ji, *J. Power Sources* 2017, 343, 76.

25. P. Liu, J. Zhou, G. Li, M. Wu, K. Tao, F. Yi, W. Zhao and L. Han, *Dalton Trans.* 2017, 46, 7388.

26. H. Du, C. Zhou, X. Xie, H. Li, W. Qi, Y. Wu and T. Liu, *Int. J. Hydrogen Energy* 2017, 42, 15236.

27. K. Wang, J. Yang, J. Zhu, L. Li, Y. Liu, C. Zhang and T. Liu, *J. Mater. Chem. A* 2017, 5, 11236.

28. G. Wei, X. Zhao, K. Du, Z. Wang, M. Liu, S. Zhang, S. Wang, J. Zhang and C. An, *Chem. Eng. J.* 2017, 326, 58.

29. Q. X. Xia, J. M. Yun, R. S. Mane, L. Li, J. Fu, J. H. Lim and K. H. Kim, *Sustainable Energy Fuels*, 2017, 1, 529.

30. S. Liu, K. S. Hui, K. N. Hui, H. Li, K. W. Ng, J. Xu, Z. Tang and S. C. Jun, *J. Mater. Chem. A* 2017, 5, 19053.

31. C. Lamiel, V. H. Nguyen, D. R. Kumar and J. Shim, *Chem. Eng. J.* 2017, 316, 1091.

32. Y. Zheng, Z. Lin, W. Chen, B. Liang, H. Du, R. Yang, X. He, Z. Tang and X. Gui, *J. Mater. Chem. A* 2017, 5, 5886.

33. B. Zhao, T. Wang, L. Jiang, K. Zhang, M. M. F. Yuen, J. Xu, X. Fu, R. Sun and C. Wong, *Electrochim. Acta* 2016, 192, 205.

34. J. Zhao, Z. Li, M. Zhang, A. Meng and Q. Li, *ACS Sustainable Chem. Eng.* 2016, 4, 3598.

35. V. Kannan, A. I. Inamdar, S. M. Pawar, H. Kim, H. Park, H. Kim, H. Im and Y. S. Chae, *ACS Appl. Mater. Interfaces* 2016, 8, 17220.

36. X. Zang, Z. Dai, J. Guo, Q. Dong, J. Yang, W. Huang and X. Dong, *Nano Res.* 2016, 9, 1358.

37. C. Long, Y. Xiao, M. Zheng, H. Hu, H. Dong, B. Lei, H. Zhang, J. Zhuang and Y. Liu, *Electrochim. Acta* 2016, 210, 695.

38. S. Abouali, M. A. Garakani. Z. Xu and J. Kim, *Carbon* 2016, 102, 262.

39. P. Wen, M. Fan, D. Yang, Y. Wang, H. Cheng and J. Wang, *J. Power Sources* 2016, 320, 28.

40. T. Li, Y. Zuo, X. Lei, N. Li, J. Liu and H. Han, *J. Mater. Chem. A* 2016, 4, 8029.

41. L. Niu, Y. Wang, F. Ruan, C. Shen, S. Shan, M. Xu, Z. Sun, C. Li, X. Liu and Y. Gong, *J. Mater. Chem. A* 2016, 4, 5669.

42. Y. Yan, P. Gu, S. Zheng, M. Zheng, H. Pang and H. Xue, *J. Mater. Chem. A* 2016, 4, 19078.

43. X. Qi, W. Zheng, X. Li and G. He, *Sci Rep.* 2016, 6, 33241.

44. M. Zheng, H. Dong, Y. Xiao, H. Hu, C. He, Y. Liang, B. Lei, L. Sun and Y. Liu, *J. Mater. Chem. A* 2017, 5, 6921.

45. S. Y. Kim, H. M. Jeong, J. H. Kwon, I. W. Ock, W. H. Suh, G. D. Stucky and J. K. Kang, *Energy Environ. Sci.* 2015, 8, 188.

46. S. Liu, S. C. Lee, U. Patil, I. Shackery, S. Kang, K. Zhang, J. H. Park, K. Y. Chung and S. C. Jun, *J. Mater. Chem. A* 2017, 5, 1043.

47. K. Qiu, M. Lu, Y. Luo and X. Du, *J. Mater. Chem. A* 2017, 5, 5820.

48. V. Senthikumar, F. B. Kadumudi, N. T. Ho, J. Kim, S. Park, J. Bae, W. M. Choi and Y. S. Kim, *J. Power Sources* 2016, 303, 363.

49. Y. Zhao, L. Hu, S. Zhao and L. Wu, *Adv. Funct. Mater.* 2016, 26, 4085.

50. W. Fu, Y. Wang, W. Han, Z. Zhang, H. Zha and E. Xie, *J. Mater. Chem. A* 2016, 4, 173.

51. J. Yang, C. Yu, X. Fan, S. Liang, S. Li, H. Huang, Z. Ling, C. Hao and J. Qiu, *Energy Environ. Sci.* 2016, 9, 1299.

52. C. Guan, W. Zhao, Y. Hu, Z. Lai, X. Li, S. Sun, H. Zhang, A. K. Cheetham and J. Wang, *Nanoscale Horiz.* 2017, 2, 99.

53. A. M. Elshhawy, C. Guan, X. Li, H. Zhang, Y. Hu, H. Wu, S. J. Pennycook and J. Wang, *Nano Energy* 2017, 39, 162.

54. Y. Guan, L. Yu, X. Wang, S. Song and X. W. Lou, *Adv. Mater.* 2017, 29, 1605051.
